# Prospects of Cinnamon in Multiple Sclerosis

**DOI:** 10.4172/2376-0389.1000149

**Published:** 2015

**Authors:** Kalipada Pahan

**Affiliations:** 1 Department of Neurological Sciences, Rush University Medical Center, Chicago, IL 60612, USA; 2 Division of Research and Development, Jesse Brown Veterans Affairs Medical Center, 820 South Damen Avenue, Chicago, IL 60612, USA

Although the central nervous system (CNS) is separated from the immune system by blood-brain barrier and traditionally considered “immune priviledged”, our immune cells are capable of targeting the brain, leading to the development of CNS autoimmune disorders. Multiple sclerosis (MS) is one such autoimmune disorder of the CNS in which myelin components are particularly targeted by the immune system resulting in demyelination of axons and associated debilitating symptoms [1–3]. In spite of intense investigations, no effective therapy is available for this disease. Therefore, a safe and effective therapeutic option is necessary for MS.

Fortunately, we have been endowed with enormous natural remedies to take care our health issues. For example, cinnamon, the brown bark of cinnamon tree, that has already been being used for centuries throughout the world as spice or flavoring agent. Furthermore, medieval physicians used cinnamon for medical purposes to treat a variety of disorders including arthritis, coughing, hoarseness, sore throats, etc. Recent studies are indicating that this natural product may be helpful for MS [4–6]. Although the etiology of MS is poorly understood, it is becoming clear that widespread inflammation, loss of regulatory T cells (Tregs), hyperactivity of autoimmune Th1 and Th17 cells, breakdown of blood-brain barrier (BBB) and blood-spinal cord barrier (BSB), and loss of neuroprotective molecules in the CNS are critical for the manifestation of demyelinating pathology in MS [1–3]. Interestingly, cinnamon treatment is capable of modifying these pathological features in mice with experimental allergic encephalomyelitis (EAE), an animal model of MS [4,6].

## Types of cinnamon

*Cinnamonum cassia* (Chinese cinnamon) and *Cinnamonum verum* or *Cinnamonum zylencum* (Ceylon cinnamon) are two readily available brands of cinnamon in the world. Although both types of cinnamon contain cinnamaldehyde as the major compound, we have seen the presence of coumarin, a hepatotoxic molecule, in *Cinnamonum cassia*, but not *Cinnamonum verum*. Furthermore, *Cinnamonum cassia* contain more styrene, benzene, 1,1′-(2-butene-1,4-diyl)bis-, benzene, 1,1′-(1,2-cyclobutanediyl)bis-, palmitic acid, stearic acid, 4-phenylbutyl chloride, and (2,3-diphenylcyclopropyl) methyl phenyl sulfoxide, which are present in *Cinnamonum verum* in negligible amounts [7]. Therefore, for the treatment of mice with EAE, we used *Cinnamonum verum*.

Upon ingestion, cinnamaldehyde present in Cinnamon is converted into cinnamic acid by oxidation. In the liver, this cinnamic acid is β-oxidized to benzoate that exists as sodium benzoate (NaB) or benzoyl-CoA. A minor amount of NaB, a direct metabolite of cinnamic acid, is also excreted in the urine of humans. Recently, we have demonstrated that oral administration of ground *Cinnamonum verum* increases the level of NaB in serum and brain of mice [7]. NaB is of medical importance as being a component of Ucephan, a FDA-approved drug, it is used in the treatment for hepatic metabolic defects associated with hyperammonemia such as urea cycle disorder in children [8,9]. NaB is also a widely used preservative in broad range of foods and cosmetic products. It has been reported that 2% solution of NaB in drinking water is safe for lifelong treatment in mice without any noticeable side effects [10].

## Attenuation of Neuroinflammation by Cinnamon Treatment

Glial activation and associated neuroinflammation plays an important role in the pathogenesis of different neuroinflammatory disorders including MS [11–13]. Interestingly, NaB treatment is capable of inhibiting the expression of proinflammatory molecules in cultured astrocytes and microglia [14]. Reversal of NaB mediated inhibition of NF-κB activation and inducible nitric oxide synthase (iNOS) expression by mevalonate, 3-hydroxy-3-methylglutaryl-coenzyme A and farnesyl pyrophosphate, intermediates of the mevalonate pathway, in activated astrocytes suggests that NaB exhibits anti-inflammatory effect via inhibiting the cholesterol-biosynthetic pathway [14]. However, although NaB is capable of reducing the level of cholesterol, cholesterol is not involved in NaB-mediated inhibition of iNOS [14]. Suppression of p21^ras^ activation by NaB Brahmachari et al., and inhibition of NF-κB activation and iNOS expression by dominant-negative mutant of p21^ras^ [15] suggest that NaB attenuates the activation of NF-κB and the expression of iNOS in glial cells via reducing the activation of p21^ras^. Accordingly, oral administration of cinnamon powder [6] and drinking water containing its metabolite NaB [4] suppress the expression of iNOS and IL-1β *in vivo* in the spinal cord and cerebellum of EAE mice, suggesting that cinnamon is capable of reducing inflammation *in vivo* in the CNS of EAE mice. Consistent to the fact that neuroinflammation is a common feature of other neurodegenerative disorders such as Alzheimer’s disease (AD) and Parkinson’s disease (PD), cinnamon and NaB also improve hippocampal functions in an animal model of AD [16] and protect the nigrostriatum in an animal model of PD [17].

## Protection of Tregs by Cinnamon Treatment

Regulatory T cells (Tregs) are regarded as the master regulator of immune responses because this cell type plays a pivotal role in the maintenance of homeostasis between immune response and immune tolerance [18,19]. Tregs suppress activation and proliferation of self-reactive T cells and thereby inhibit immune response of self-reactive T cells against self-antigens [19,20]. The transcription factor Foxp3 and the surface protein CD25 are the two key molecules characterizing Tregs. Recent studies suggest that the expression of Foxp3 and the numbers of peripheral CD4^+^CD25^+^ Foxp3^+^ T cells are significantly reduced in relapsing-remitting MS patients compared with those in control subjects [18]. Therefore, up regulation and/or maintenance of Tregs may be beneficial for MS. We have delineated that oral feeding of cinnamon powder is capable of enriching Foxp3^+^ Tregs *in vivo* in mice [16]. Foxp3^+^ Tregs are also characterized by CD25, CD62L, CTLA4, GITR etc. [19]. Accordingly, cinnamon treatment inhibited the loss of CD25, CD62L, CTLA4, and GITR in the spleen of EAE mice [6]. Consistent to the suppressive activity of Tregs, cinnamon-induced Tregs also inhibit the level of IFN-γ in autoreactive T cells [6].

While investigating mechanisms behind cinnamon-mediated protection of Tregs, we have seen that blocking NO either by inhibiting iNOS or direct scavenging of NO or by pharmacological drugs restores the expression of Foxp3 in MBP-primed T cells [21]. Furthermore, NO donors decrease Foxp3 [21] and cinnamon treatment enriches Tregs via suppressing NO production [6]. Since cinnamon is metabolized to NaB and NaB attenuates the production of NO [14] and restores Foxp3 in MBP-primed splenocytes via suppression of NO [21], it is likely that cinnamon is metabolized to NaB, which then suppresses the production of NO in spleen to protect Tregs in EAE mice.

## Suppression of Th1 and Th17 Cells by Cinnamon Treatment

Earlier MS was considered mainly a Th1-mediated autoimmune disease [22]. However, since the discovery of IL-23 in 2000 [23], Th17 cells are considered to play a more active role than Th1 cells in the disease process of EAE and MS [24]. It is believed that autoreactive and inflammatory population of Th1 and Th17 cells participates in the demyelination of CNS white matter [22,24]. Therefore, suppressing Th17 and Th1 cells may be beneficial for MS and EAE. Th17 cells are driven by a transcription factor called RORγt [25]. Interestingly, oral administration of cinnamon markedly suppressed EAE-induced up regulation of IL-17 and RORγt mRNAs as well as the CD4+IL-17+ and CD4+RORγT+ Th17 cell population [6]. While T-bet-dependent IFN-γ production is a characteristic of Th1 cells, Th2 cells display GATA3-dependent IL-10 and IL-4 release [5,26]. Treatment of EAE mice with cinnamon led to the suppression of IFN-γ and T-bet mRNAs as well as the CD4+IFN-γ+ Th1 cells, while augmenting the CD4+IL-4+ Th2 response [6]. Since Tregs are known to suppress the proliferation of autoimmune Th1 cells by secreting TGF-β and IL-10 and of autoimmune Th17 cells via release of IL-35, it is possible that cinnamon treatment suppresses Th17 and Th1 responses in EAE mice via Tregs.

## Maintenance of BBB and BSB Integrities by Cinnamon Treatment

BBB and BSB are membranic permeability barriers that act primarily to protect the brain and the spinal cord respectively from chemicals in the blood, while still allowing some essential molecules such as glucose to enter. It is known that during active MS and EAE, BBB and BSB break down in a section of the brain and spinal cord respectively due to widespread inflammation, thereby allowing different blood molecules and toxins enter into the CNS. Accordingly, significant amount of infra-red dye is visible in CNS tissues of EAE mice [6]. However, oral administration of cinnamon markedly attenuates the entry of infra-red dye into the spinal cord and different parts of the brain, suggesting that cinnamon treatment preserves the integrity of BBB and BSB in EAE mice. Accordingly, cinnamon treatment also reduced infiltration and the appearance of cuffed vessels in spinal cord of EAE mice [6]. Attenuation of different cell adhesion molecules and integrins in the CNS of EAE mice by NaB treatment [7] suggests that cinnamon-mediated restoration of BBB and BSB integrities is probably mediated by NaB.

## Cinnamon and Demyelination

**Figure 1:**
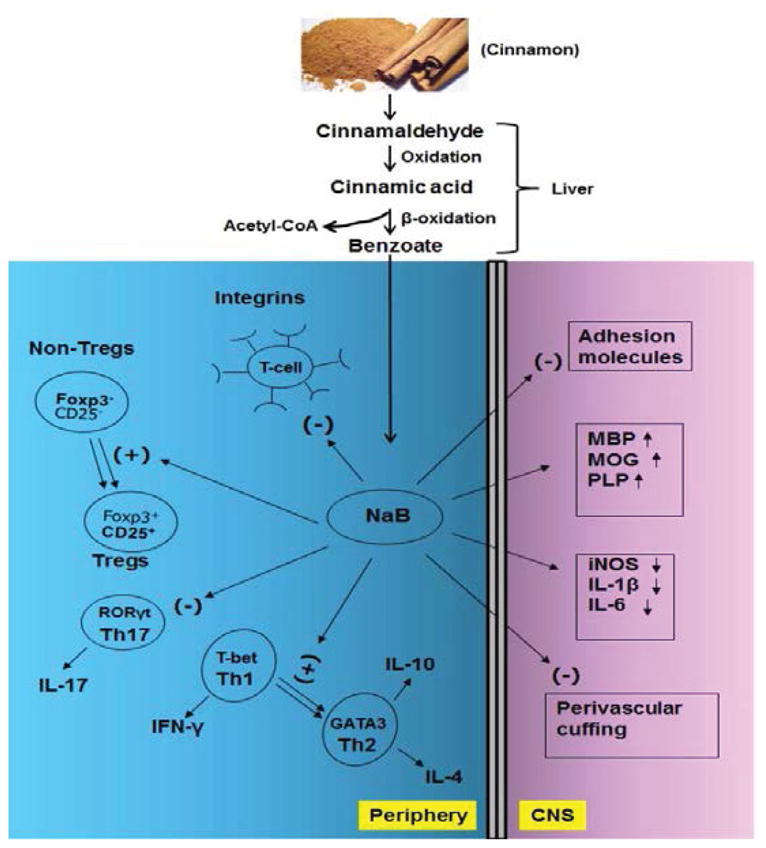
Schematic diagram representing the effect of cinnamon on autoimmune demyelination. The major component of cinnamon is cinnamaldehyde, which is oxidized to cinnamic acid. This cinnamic acid then undergoes β-oxidation to be converted into benzoate that exists as sodium benzoate (NaB). In the periphery, NaB inhibits several integrins on T cells, enriches Tregs, suppresses Th17 cells, and causes Th1 to Th2 shift. On the other hand, NaB treatment leads to suppression of adhesion molecules, attenuation of mononuclear cell infiltration, decrease in proinflammatory molecules, and normalization and/or up regulation of myelin genes in the CNS compartment.

MS symptoms develop due to demyelination, resulting in inability of nerves to conduct electrical impulses to and from the brain. Although precise mechanism leading to demyelination in MS patients is not known, it is believed that breakdown of BBB and BSB, infiltration of blood mononuclear cells and associated neuroinflammation play an important role in CNS demyelination observed in MS patients and EAE animals [1–2]. Accordingly, widespread demyelination zones and loss of myelin specific genes (myelin basic protein, MBP; proteolipid protein, PLP; myelin oligodendrocyte glycoprotein, MOG) in spinal cord white matter are observed of mice with both relapsing remitting (RR) EAE and chronic (Ch) EAE [6]. However, cinnamon treatment remarkably restored myelin level and protected myelin-specific genes in the spinal cord of mice with RR-EAE and Ch-EAE [6] (Figure 1).

## Cinnamon and Clinical Symptoms of EAE

Since cinnamon and its metabolite NaB reduce glial inflammation, upregulate Tregs, suppress Th17 and Th1 cells, inhibit inflammatory infiltration, restore the integrity of BBB and BSB, and protect myelin, the therapeutic efficacy of cinnamon and NaB was tested in EAE mice. NaB, administered through drinking water at physiologically tolerable doses, ameliorated clinical symptoms and disease progression of EAE in recipient mice and suppressed the generation of encephalitogenic T cells in donor mice [4]. Accordingly, oral cinnamon treatment improved locomotor activities and inhibited clinical symptoms of RR-EAE in female PLP-TCR transgenic mice and adoptive transfer mouse model. Cinnamon also reduced clinical symptoms of chronic EAE in male C57/BL6 mice [6].

Tysabri and different forms of interferon-β (IFN-β) are currently used to treat MS [27,28]. However, reduced effectiveness and severe toxic effects over chronic use, as well as treatment costs, often limit these available therapies. For example, IFN-β taken through painful injections has a number of side effects including flu-like symptoms, menstrual disorders in women, decrease in neutrophil and white blood cell count, increase in aspartate transaminase (AST) and alanine transaminase (ALT) levels, and development of neutralizing antibodies to IFN-β [3,29,30]. Similarly, treatment with Tysabri can cause lung infection, breathing problems, chest pain, wheezing, urinary tract infection, vaginitis, nausea, vomiting, and liver damage. Tysabri also increases the chance of getting a severe brain infection, leading to progressive multifocal encephalopathy, which may cause disability and death [28,31].

On the other hand, cinnamon has a long track record as a non-toxic natural product. It can be taken orally, the least painful route. After oral administration, cinnamon is metabolized into NaB, the active compound, in the liver, which then readily enters into the brain [7]. Similarly, the metabolite NaB also can be taken through food and drinking water or milk. However, cinnamon, being a more economical and natural option than NaB, fits well for therapeutic intervention in MS. However, before recommending cinnamon to MS patients, it is important to know the long-term effect of cinnamon and NaB in different animal models of MS. Finally, a clinical trial either as an add-on with Betaseron^®^ or monotherapy is needed to authenticate the aroma of cinnamon in MS.
